# Diagnostic Performance of Standardized First Trimester Fetal Echocardiography for the Detection of Congenital Heart Defects

**DOI:** 10.1002/jcu.70016

**Published:** 2025-07-19

**Authors:** Münip Akalın, Emine Eda Akalın, Merve Kara, Esra Esim Büyükbayrak

**Affiliations:** ^1^ Department of Perinatology Marmara University Pendik Training and Research Hospital Istanbul Turkey; ^2^ Department of Obstetrics and Gynaecology Bahcesehir University School of Medicine Istanbul Turkey; ^3^ Department of Obstetrics and Gynaecology Marmara University Pendik Training and Research Hospital Istanbul Turkey

**Keywords:** cardiac ultrasound, congenital heart defects, fetal echocardiography, first trimester, ultrasound

## Abstract

**Purpose:**

To determine the diagnostic accuracy of standardized first trimester (11^+0^–14^+0^ weeks of gestation) fetal echocardiography (FE) for the detection of congenital heart defects (CHDs).

**Methods:**

Pregnant women who underwent FE in the first trimester at a tertiary center between November 2021 and December 2022 were included in this retrospective study. In the first trimester, FE; heart position with cardiac axis, heart rhythm, four‐chamber view with two distinct ventricles, left ventricular outflow tract view, and three vessel trachea view were evaluated. CHDs were grouped as mild (do not require treatment or can be easily treated without affecting the child in the long term) and severe (expected to require surgery or cardiac catheterization, may affect the child in the long term) according to their severity.

**Results:**

A total of 311 pregnant women were included in the study, and 22 (7.1%) fetuses were diagnosed with CHDs. Of the 22 fetuses, 6 (27.3%) had severe CHDs, and 16 (72.7%) had mild CHDs. All severe CHDs were correctly diagnosed on first trimester FE. The sensitivity of first trimester FE for overall CHDs was 54.5%, and specificity was 100%. The performance of first trimester FE in the diagnosis of severe CHDs was excellent, with 100% sensitivity and 100% specificity.

**Conclusion:**

First trimester FE has high detection rates for early diagnosis of severe CHDs. Standardized first trimester FE may allow early diagnosis of fetuses with CHDs and contribute to the appropriate management of these pregnancies.

## Graphical abstract

First trimester fetal echocardiography has high detection rates for early diagnosis of severe congenital heart defects. Standardized first trimester fetal echocardiography may allow early diagnosis of fetuses with congenital heart defects and contribute to the appropriate management of these pregnancies.

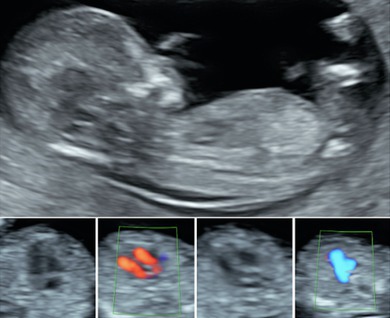

## 1 Introduction

Congenital heart defects (CHDs) are one of the most common fetal anomalies, with an incidence of 9 per 1000 live births (van der Linde et al. 2011). Although a significant portion of CHDs are minor anomalies, approximately 3 in 1000 live births have severe CHDs and are one of the leading causes of death due to fetal anomalies (Karim et al. 2022). Prenatal diagnosis of CHDs can reduce disease‐related morbidity and mortality by providing appropriate prenatal care to patients and planning their birth in reference centers (Fuchs et al. 2007). Additionally, prenatal diagnosis may enable invasive genetic diagnostic testing to diagnose possible fetal genetic abnormalities and allow parents to be offered the option of terminating a pregnancy in fetuses with severe CHDs.

The majority of CHDs occur in fetuses without identifiable high‐risk factors, and therefore, without widespread screening in the low‐risk population, most CHDs remain undiagnosed (Sharland 2004). For this reason, in many countries, fetal heart examination is performed as part of routine second trimester ultrasonography screening. On the other hand, advances in ultrasonography technology have enabled the fetal heart to be examined for CHDs in the first trimester. However, there is no international consensus on how to perform a first trimester fetal heart examination. Recommendations range from simply confirming a heartbeat to a detailed examination that includes routine four‐chamber view with outflow tract imaging (Karim et al. 2022; International Society of Ultrasound in Obstetrics and Gynecology 2023; Khalil and Nicolaides 2013). As expected, detailed examination of fetal heart anatomy increases the detection rate of CHDs (Karim et al. 2022). However, in the last decade, there have been few studies evaluating the performance of detailed cardiac examination in low‐risk populations (Bottelli et al. 2023; Syngelaki et al. 2019; Duta et al. 2021; Vayna et al. 2018; Zheng et al. 2019; Chen et al. 2019; Ebrashy et al. 2019). In addition, there is limited data on the contribution of indirect findings that may be related to CHDs, such as nuchal translucency (NT) thickness, abnormal ductus venosus flow, and hydrops fetalis, to the diagnosis of CHDs.

The aim of this study was to determine the diagnostic accuracy of standardized detailed first trimester fetal echocardiography (FE) in the detection of CHDs and to investigate the contribution of indirect findings to the diagnosis.

## 2 Materials and Methods

Pregnant women who underwent fetal ultrasonographic screening and fetal cardiac examination in the first trimester between November 2021 and December 2022 in our tertiary center were included in this retrospective study. The study was approved by the ethics committee of our institute (approval number: 09.2023.91). Patient data were obtained retrospectively from our center's patient data recording system. First trimester ultrasonographic examinations were performed between 11^+0^ and 14^+0^ weeks of gestation. Patients whose fetal cardiac examination could not be performed in the first trimester, whose pregnancy was terminated in the first trimester due to fetal genetic anomaly or severe fetal malformation, and whose second trimester ultrasonographic screening was not performed in our center were excluded from the study.

Fetal anatomical examination in the first trimester ultrasonographic screening was performed according to published guidelines (International Society of Ultrasound in Obstetrics and Gynecology 2023; Volpe et al. 2016). Crown‐rump length (CRL) and NT were measured on the mid‐sagittal section of the fetal head and neck obtained by 2D ultrasonography as previously described (Nicolaides 2004). In the first trimester fetal heart examination, heart position with cardiac axis to the left (30°–60°) and situs, heart rhythm, four‐chamber view on grayscale and color Doppler, left and right ventricular outflow tract view on grayscale and color Doppler, three‐vessel and trachea view, and right subclavian artery view on grayscale and color Doppler were evaluated. Anomalies detected in fetal heart examination were determined as direct findings of CHDs. Abnormal cardiac axis, NT thickness (≥ 3 mm), abnormal venous flow pattern in the ductus venosus (absence or reverse of a‐wave), and hydrops fetalis were determined as indirect findings for CHDs. In the second trimester ultrasonography, fetal anatomical screening and FE were performed between 18^+0^ and 24^+0^ weeks of gestation according to published guidelines (Salomon et al. 2022; International Society of Ultrasound in Obstetrics and Gynecology et al. 2013; AIUM 2020). In the second trimester fetal heart examination, cardiac position and axis, heart rhythm, ventricles and atria in four‐chamber view, atrioventricular valves, interventricular and interatrial septum, pulmonary venous return, left and right ventricular outflow tracts, three‐vessel and trachea view, aortic and ductal arches, and systemic venous return were evaluated by grayscale and color Doppler examination. Fetal ultrasonography and fetal measurements were performed with a GE Voluson E6 ultrasonography device with a 4–8 MHz transabdominal probe. If a suitable image could not be obtained with the transabdominal probe in the first trimester ultrasonography, transvaginal ultrasonography was performed with a 5–10 MHz endocavitary probe.

Maternal demographic characteristics, fetal ultrasonography reports, and first trimester combined test results of the patients were recorded. Maternal chronic diseases were defined as autoimmune diseases, endocrinological diseases, chronic hypertension, hematological diseases, and neurological diseases. Gestational ages were calculated according to the CRL measurement in the first trimester, and the gestational ages in the first trimester ultrasonography and second trimester ultrasonography were recorded.

CHDs were grouped as mild (do not require treatment such as persisted left superior vena cava [PLSVC] and aberrant right subclavian artery [ARSA], or can be easily treated without affecting the child in the long term such as ventricular septal defect [VSD]) and severe (expected to require surgery or cardiac catheterization, may affect the child in the long term after birth such as atrioventricular septal defect [AVSD], transposition of the great arteries [TGA], and double outlet right ventricle [DORV]) according to their severity. The diagnosis of all fetuses diagnosed with CHDs during the antenatal period was confirmed by postnatal echocardiography. The performance of first trimester ultrasonography in the diagnosis of mild and severe CHDs was evaluated.

### 2.1 Statistical Analyses

Descriptive statistics were reported as frequencies (%), in addition to the mean ± standard deviation. The Kolmogorov–Smirnov test was used to evaluate the distribution of continuous variables. The Mann–Whitney *U* test was applied to explore relationships among non‐categorical variables. For assessing the statistical significance of categorical variables, either Pearson's Chi‐square test or Fisher's exact test was conducted. Indirect findings associated with CHDs were tested by univariate analysis. In logistic regression analysis, possible indirect findings related to CHDs identified in univariate analysis were included in the analysis. Sensitivity, specificity, negative predictive values (NPV) and positive predictive values (PPV) were calculated for first trimester FE and indirect findings in the diagnosis of CHDs. The data analysis was performed using the SPSS software version 21 package program. Statistical significance was defined as *p* ≤ 0.05.

## 3 Results

**FIGURE 1:**
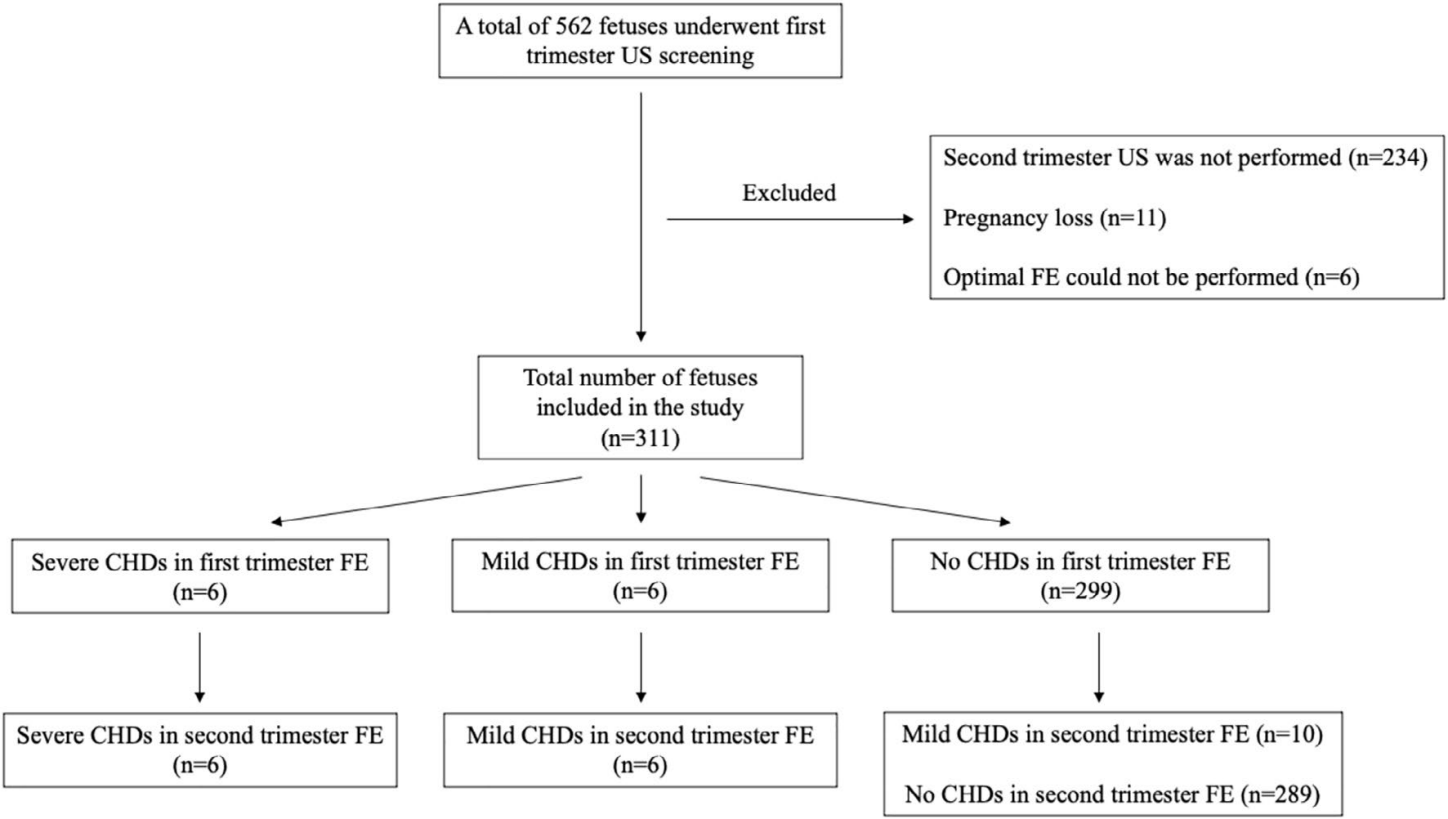
Flow chart of fetuses included in the study. CHDs, congenital heart defects; FE, fetal echocardiography; US, ultrasonography.

During the study period, first trimester ultrasonography was performed on a total of 562 fetuses. The flow chart of the cases included in the study is shown in Figure 1. Eleven (1.9%) patients with pregnancy loss, 6 (1.1%) patients in whom optimal first trimester FE could not be performed, and 234 (41.6%) patients who did not undergo second trimester ultrasonography were excluded from the study. The baseline characteristics of the 311 patients included in the study were shown in Table 1.

**TABLE 1:** The baseline characteristics of the patients included in the study.

	Patients with fetal CHDs (*n* = 22)	Patients without fetal CHDs (*n* = 289)	*p* values
Maternal age (years)	31.2 ± 4.1	30.2 ± 5.1	0.30
Maternal BMI (kg/m^2^)	25.8 ± 3.5	25.5 ± 5.2	0.50
Gravida	2.7 ± 1.2	2.5 ± 1.5	0.19
Parity	1.1 ± 1.0	1.1 ± 1.0	0.72
Maternal active chronic disease	5 (22.7)	110 (38.1)	0.15
GA at first trimester FE (weeks)	12.7 ± 0.7	12.4 ± 0.6	**0.02**
CRL (mm)	68.4 ± 7.6	63.9 ± 7.8	**0.01**
NT (mm)	3.2 ± 2.4	1.7 ± 0.7	**0.003**
GA at second trimester FE (weeks)	20.6 ± 1.6	21.3 ± 1.7	**0.03**
EFW on second trimester FE (g)	403.3 ± 82.3	476.6 ± 230.1	**0.02**
Non‐cardiac fetal anomaly	1 (4.5)	10 (3.5)	0.56

*Note*: Values are expressed as mean ± standard deviation, number, and percentage (%). *p*‐values marked with bold indicate statistically significant differences between the groups.

Abbreviations: BMI, body mass index; CHDs, congenital heart defects; CRL, crown–rump length; EFW, estimated fetal weight; FE, fetal echocardiography; GA, gestational age; NT, nuchal translucency.

**FIGURE 2:**
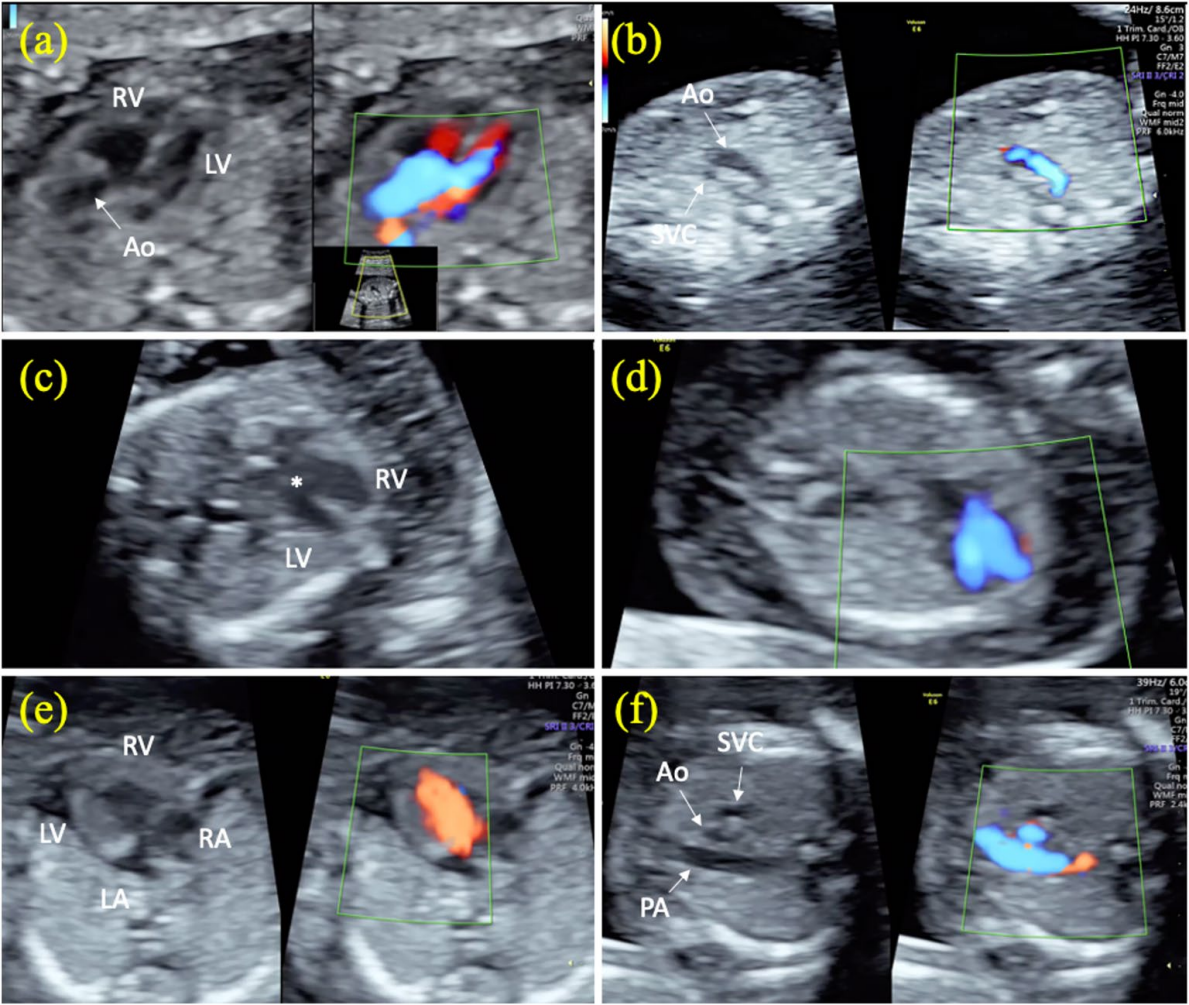
First trimester fetal echocardiographic images of tetralogy of Fallot, atrioventricular septal defect, and hypoplastic left heart syndrome. Tetralogy of Fallot (a, b). Note the course of the aorta in the left ventricular outflow tract (overriding aorta) and color Doppler image in the same section (a). There are two vessels (aorta and superior vena cava) in the 3‐vessel view of the same fetus (b). Atrioventricular septal defect (c, d). Asterisk indicates endocardial cushion defect in gray scale (c) and ventricular filling on color Doppler (d). Hypoplastic left heart syndrome (e, f). Appearance of left ventricular hypoplasia in gray scale and color Doppler (e). Note that the aorta is hypoplastic in the 3‐vessel view in the same fetus (f). Ao, aorta; LA, left atrium; LV, left ventricle; PA, pulmonary artery; RA, right atrium; RV, right ventricle; SVC, superior vena cava.

**FIGURE 3:**
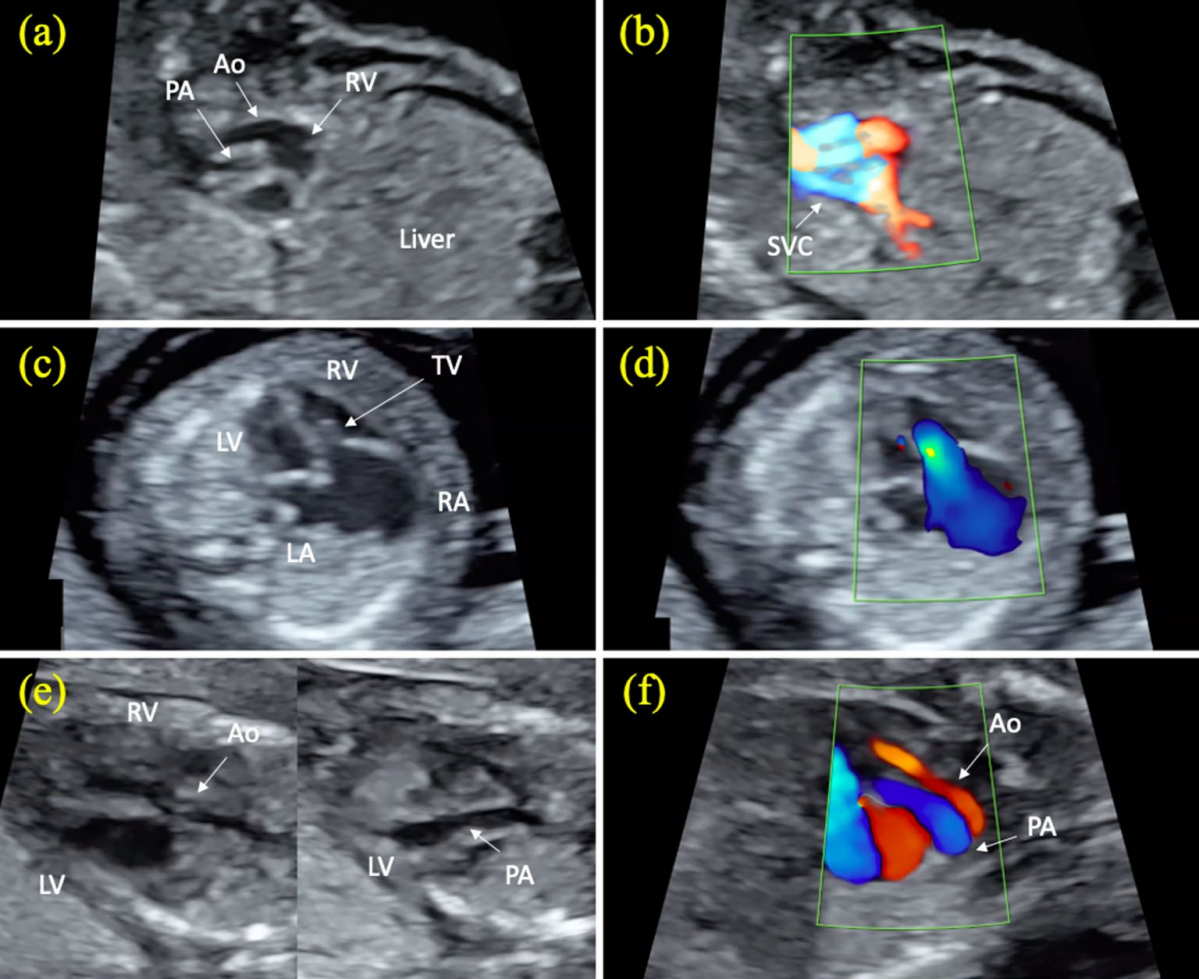
First trimester fetal echocardiographic images of double outlet right ventricle, Ebstein anomaly, and transposition of the great arteries. Double outlet right ventricle (a, b). Gray scale (a) and color Doppler (b) view of the aorta and pulmonary artery originating from the right ventricle. Note that the aorta and pulmonary artery are not crossed. Ebstein anomaly (c, d). In gray scale, the tricuspid valve is located more apically in the four‐chamber view, and the left atrium is dilated (c). Severe tricuspid regurgitation with apical onset on color Doppler in the same fetus (d). Transposition of the great arteries (e, f). In gray scale, the aorta originates from the right ventricle, and the pulmonary artery originates from the left ventricle (e). Color Doppler image of the parallel course of the aorta and pulmonary artery in the sagittal oblique section in the same fetus (f). The aortic valve is dysplastic, and there is reverse flow in the aorta. Ao, aorta; LA, left atrium; LV, left ventricle; PA, pulmonary artery; RA, right atrium; RV, right ventricle; SVC, superior vena cava; TV, tricuspid valve.

A total of 22 (7.1%) fetuses were diagnosed with CHDs. Of the 22 fetuses, 6 (27.3%) had severe CHDs (1 fetus with AVSD, 1 fetus with hypoplastic left heart syndrome [HLHS], 1 fetus with DORV, 1 fetus with TGA, 1 fetus with Tetralogy of Fallot [TOF], and 1 fetus with Ebstein anomaly) and 16 (72.7%) had mild CHDs (12 fetuses with VSD, 3 fetuses with ARSA, and 1 fetus with PLSVC). First trimester FE images of fetuses with severe CHDs are shown in Figures 2 and 3. All severe CHDs were correctly diagnosed on first trimester FE. Six (37.5%) of 16 fetuses with mild CHDs (3 fetuses with VSD and 3 fetuses with ARSA) were diagnosed by first trimester FE. Mild CHDs were detected in 10 (3.3%) (9 fetuses with VSD and 1 fetus with PLSVC) of 299 fetuses with normal first trimester FE. Severe CHDs were not detected in any of the fetuses with normal first trimester FE.

In the first trimester ultrasonography, 15 (4.8%) of the fetuses had NT thickness, 9 (2.9%) had hydrops fetalis, 8 (2.6%) had abnormal ductus venosus flow, and 4 (1.3%) had abnormal cardiac axis. The rates of indirect findings on first trimester ultrasonography in fetuses with mild CHDs and severe CHDs are shown in Table 2. Fetuses with CHDs had higher rates of NT thickness (36.4% vs. 2.4%, *p* < 0.0001), hydrops fetalis (22.7% vs. 1.4%, *p* < 0.0001), abnormal ductus venosus flow (22.7% vs. 1.0%, *p* < 0.0001), and abnormal cardiac axis (13.6% vs. 0.3%, *p* = 0.001) compared to those without CHDs. In logistic regression analysis, NT thickness (RR: 9.5, 95% CI: 1.2–74.5, *p* = 0.03) and abnormal ductus venosus flow (RR: 9.7, 95% CI: 1.0–91.3, *p* = 0.04) were associated with CHDs.

**TABLE 2:** The rates of indirect findings on first trimester ultrasonography in fetuses with mild CHDs and severe CHDs.

First trimester ultrasonographic findings	Fetuses without CHDs (*n* = 289)	Fetuses with mild CHDs (*n* = 16)	Fetuses with severe CHDs (*n* = 6)	*p* values
NT thickness (≥ 3 mm)	7 (2.4)	3 (18.8)	5 (83.3)	**< 0.001**
Abnormal cardiac axis	1 (0.3)	1 (6.3)	2 (33.3)	**< 0.001**
Absence/reverse a‐wave in DV	3 (1.0)	3 (18.8)	2 (33.3)	**< 0.001**
Hydrops fetalis	4 (1.4)	3 (18.8)	2 (33.3)	**< 0.001**

*Note*: Values are expressed as number and percentage (%). *p*‐values marked with bold indicate statistically significant differences between the groups.

Abbreviations: CHDs, congenital heart defects; DV, ductus venosus; NT, nuchal translucency.

The sensitivity of first trimester FE for overall CHDs (mild CHDs and severe CHDs) was 54.5%, specificity was 100%, PPV was 100%, and NPV value was 96.6%. The performance of first trimester FE in the diagnosis of severe CHDs was excellent, with 100% sensitivity and 100% specificity. However, the performance of first trimester FE in the diagnosis of mild CHDs was low (sensitivity: 37.5%, specificity: 100%, PPV: 100%, and NPV: 96.7%). The sensitivities, specificities, PPVs, and NPVs of first trimester FE and first trimester indirect ultrasonographic findings in the diagnosis of severe CHDs are shown in Table 3. The most valuable indirect finding in the diagnosis of severe CHDs was increased NT thickness, with 83.3% sensitivity and 96.7% specificity.

**TABLE 3:** The sensitivities, specificities, PPVs and NPVs of first trimester FE and first trimester indirect ultrasonographic findings in the diagnosis of severe CHDs.

	Sensitivity (%)	Specificity (%)	PPV (%)	NPV (%)
NT thickness (≥ 3 mm)	83.3	96.7	33.3	99.7
Abnormal cardiac axis	33.3	99.3	50.0	98.7
Absence/reverse a‐wave in DV	33.3	98.0	25.0	98.7
Hydrops fetalis	33.3	97.7	22.2	98.7
At least one of the indirect findings of CHDs	83.3	96.0	29.4	99.7

Abbreviations: CHDs, congenital heart defects; DV, ductus venosus; FE, fetal echocardiography; NPV, negative predictive value; NT, nuchal translucency; PPV, positive predictive value.

One (4.5%) of the fetuses with CHDs had an omphalocele as a non‐cardiac anomaly. Of the fetuses without CHDs, 10 (3.5%) had non‐cardiac anomalies (3 fetuses with central nervous system anomalies, 4 fetuses with urinary system anomalies, 2 fetuses with skeletal dysplasia, and 1 fetus with omphalocele).

## 4 Discussion

The present study comprehensively evaluated the performance of standardized first trimester FE in detecting CHDs. Our results revealed that severe CHDs can be detected with high accuracy by first trimester FE and that standardized cardiac examination on first trimester FE is reassuring in excluding the majority of severe CHDs. On the other hand, first trimester FE had a relatively poor performance in detecting mild CHDs. This finding supports that normal first trimester FE cannot completely exclude possible minor CHDs. Therefore, even if FE is normal in the first trimester, fetal heart examination in the second trimester still remains important. Secondly, our study investigated the contribution of indirect findings in the first trimester to the diagnosis of CHDs and showed that indirect findings are seen at a higher rate in fetuses with CHDs. Therefore, we think that fetal heart examination should be performed more carefully in fetuses with indirect findings, especially in fetuses with NT thickness or abnormal ductus venosus flow. Furthermore, the higher rate of CHDs in fetuses with indirect findings suggests that a screening strategy based on the presence of indirect findings may be rational in countries with limited resources.

The diagnostic accuracy of first trimester FE has been investigated in previous studies (Karim et al. 2022; Bottelli et al. 2023; Syngelaki et al. 2019; Duta et al. 2021; Vayna et al. 2018; Zheng et al. 2019; Chen et al. 2019; Ebrashy et al. 2019; Yu et al. 2020). In a meta‐analysis, the detection rate of major CHDs by first trimester FE in a non‐high‐risk population was 63.7%, and the pooled sensitivity of first trimester FE was 55.80%, specificity was 99.98%, and PPV was 94.85% (Karim et al. 2022). In another meta‐analysis, the pooled sensitivity, specificity, positive likelihood ratio, and negative likelihood ratio of first trimester FE in diagnosing major CHDs were 0.838 (95% CI, 0.795–0.875), 1.000 (95% CI, 1.000–1.000), 725.69 (95% CI, 312.562–1684.9), and 0.203 (95% CI, 0.160–0.259), respectively (Yu et al. 2020). The reason for the higher detection rate of major CHDs in our study may be due to the routine evaluation of ventricular outflow tracts and the use of color Doppler in cardiac examination. In major CHDs such as TOF and TGA, the four‐chamber view may be normal, and the diagnosis of these anomalies can only be made by evaluating the ventricular outflow tract. Therefore, we think that it is important to evaluate the ventricular outflow tracts as well as the four‐chamber view in the first trimester for the diagnosis of major CHDs.

The results of the present study showed that severe CHDs can be diagnosed with high accuracy by first trimester FE. On the other hand, some severe CHDs, such as coarctation of the aorta, may occur in advanced gestational ages (Akalın et al. 2022). Additionally, anomalies such as abnormal pulmonary venous return, partial AVSD, and interrupted aortic arch may be difficult to evaluate in first trimester FE. Therefore, a normal first trimester FE cannot exclude all severe CHDs. Additionally, our study revealed that first trimester FE sensitivity is low in mild CHDs and that these anomalies may be overlooked in the first trimester. For these reasons, we recommend re‐evaluation of the fetal heart in the second trimester, even if first trimester FE is normal.

The current study has some limitations. There were relatively few cases of major CHDs in the study, and the accuracy of first trimester FE in detecting major CHDs may have been lower in a larger number of cases. Additionally, some of the minor CHDs may not have been diagnosed on second trimester ultrasonography. Therefore, a study design that evaluates postnatal echocardiographic findings may more accurately evaluate the performance of first and second trimester cardiac examination. Lastly, in this study, all first trimester FE was performed with a high‐resolution ultrasonography device, and therefore, sensitivity and specificity may be lower in FE performed with lower resolution devices.

## 5 Conclusion

Standardized fetal cardiac examination in the first trimester may be useful in the early diagnosis of CHDs in low‐risk populations. First trimester FE has high detection rates for early diagnosis of severe CHDs. On the other hand, fetal heart examination in the second trimester remains important, as first trimester FE has a relatively poor performance in detecting mild CHDs. Although a normal first trimester cardiac examination is reassuring in excluding the vast majority of severe CHDs, second and even third trimester fetal heart examination remains valuable for the diagnosis of CHDs.

## Ethics Statement

The study was approved by the ethics committee of our institute (approval number: 09.2023.91).

## Conflicts of Interest

The authors declare no conflicts of interest.

## Data Availability

The data that support the findings of this study are available from the corresponding author upon reasonable request.
